# 

**Published:** 1860-12

**Authors:** 


### CONTENTS FOR DECEMBER, 1860.

### ORIGINAL COMMUNICATIONS.

Valedictory Address, delivered to the Medical Class in Lind University. By Professor T. DEVILLE.......... 705

Cases in Practice. By F. K. BAILEY, M.D., Joliet, Ill.......... 716

Cases in Obstetrical Practice. By B. WOODWARD, M.D., Galesburg, Ill.......... 723

A Case of Meningial Apoplexy ; Post-Mortem Appearances. Reported by Dr. HENRY LYSTER, Brooklyn, Mich.......... 727

Inversion of the Uterus. Case Reported to the Chicago Medicai Society. By IRA HATCH, M.D. (Published by Request.).......... 732

Coroner's Investigation.—Suspected Poisoning.—Case of MARY E. ROSELL, of Binghamton, N. Y... ....... 736

Correspondence.—Diphtheria.......... 742

Proceedings of the De Witt County Medical Society. Reported By CHRISTOPHER GOODBRAKE, M.D., Secretary.......... 744

Chicago Medical Society.......... 746

Clinical Reports.—Mercy Hospital. Service of Prof. N. S. DAVIS, M. D., Professor of Practical Medicine and of Clinical Medicine. (Collated by FRANK W. REILLY, Sen. Class Med. Depart. Lind University.).......... 747

Service of Prof. E. ANDREWS, M.D., Professor of Surgery in the Medical Depart. Lind University.......... 751

### BOOK AND PAMPHLET NOTICES.

Transactions of the Illinois State Medical Society, at its Tenth Annual Meeting, held in Paris, May 8th and 9th, 1860.......... 756

Transactions of the Indiana State Medical Society, at its Eleventh Annual Session, held in Indianapolis, May 17th and 18th, 1860.......... 756

To what Affections of the Lungs does Bronchitis give Origin? An Essay. By DANIEL D. SLADE, M.D., of Boston.......... 757

Annual Address, delivered before the Philadelphia Medical Society, March 6th, 1860. By R. LaROCHE, M.D.......... 760

Contributions to the Medical Flora of Nashville, Tenn. By GEO. S. BLACKIE, M.D. 760

Medico-Legal Inquiry. By T. L. WRIGHT, M. D., of Bellefontaine, Ohio.......... 760

### SELECTIONS.

Tapeworm.......... 661
M. Jobert's Method of Treating Stricture.......... 762
Cold vs. Heat.......... 762
The Cholera in Spain.......... 762
Ethereal Instillations into the Ear for the Cure of Deafness.......... 763
Effectual Use of the Sponge Tent in Sterility.......... 763
Smokers and non-Smokers in the Polytechnic School of Paris.......... 763
A Hospital for Negroes at Charleston, S. C.......... 763
JOHN HUNTER.......... 764

### EDITORIAL.

A Request.......... 764
Fisher vs. Stone.......... 765
Resignation.......... 766
The Examiner.......... 767
Medical Department of Lind University.......... 767

